# Supplementary material for: How the persistence of patriarchy undermines the financial empowerment of women microfinance borrowers? Evidence from a southern sub-district of Bangladesh
Source: PLoS One. 2021 Apr 28;16(4):e0250000. doi: 10.1371/journal.pone.0250000 (PMC8081197; doi:10.1371/journal.pone.0250000)
Supplement: Attachment — Submitted filename: Reviewer.doc [file pone.0250000.s001.doc]

Reviewer-1	Comments

1. The citations format allows to mention the author name and respective governments are considered the author of all the official reports; rather mentioning the name of particular organization. For example “Microfinance Regulatory Authority, 2018 at Pg-6”.
2. The specified characteristics defined for the baseline to identify the informants of this study may lead to create a biases as well as rigid sample and in the presence of such sample it becomes difficult to generalize the results for the entire women borrowing community.
3. Because of the fact that the study based upon the primary information and thus it is imperative to employ suitable statistical techniques i.e. Cronbach's Alpha, to examine the reliability of data.
4. In the presence of such a rich information, the use of regression analysis may be a good addition and also more interesting; rather using the simple percentages. Hence, it is suggested to use an appropriate econometric model i.e. Logit or Probit, to empirically determine the facts & figure for the policy derivation.
5. No doubt the study is very interesting, but it lacks the suggested remedial measures to overcome this issue.
