# Supplementary material for: How the persistence of patriarchy undermines the financial empowerment of women microfinance borrowers? Evidence from a southern sub-district of Bangladesh
Source: PLoS One. 2021 Apr 28;16(4):e0250000. doi: 10.1371/journal.pone.0250000 (PMC8081197; doi:10.1371/journal.pone.0250000)
Supplement: Attachment — Submitted filename: Responses to Reviewers_PONE-D-20-29072.docx [file pone.0250000.s002.docx]

| Dear editor,  Please find the rebuttal letter for the manuscript titled “How the persistence of patriarchy undermines the financial empowerment of women microfinance borrowers? Evidence from a southern sub-district of Bangladesh” (followed by) that responds to each point raised by the academic editor and reviewers.  We thank you all for your valuable comments and suggestions made to improve our submission. Please let us know if there anything more we need do to answer your queries.  Tunvir Ahamed Shohel (corresponding author)  Sociology Discipline  Khulna University, Bangladesh  Email: [tunvirshohel@ku.ac.bd](mailto:tunvirshohel@ku.ac.bd) | | |
| --- | --- | --- |
| Editor | **Comments** | **Authors’ response** |
|  | A rebuttal letter that responds to each point raised by the academic editor and reviewer(s). You should upload this letter as a separate file labeled 'Response to Reviewers'. | *Uploaded* |
|  | A marked-up copy of your manuscript that highlights changes made to the original version. You should upload this as a separate file labeled 'Revised Manuscript with Track Changes'. | *Uploaded* |
|  | An unmarked version of your revised paper without tracked changes. You should upload this as a separate file labeled 'Manuscript'. | *Uploaded* |
|  | Please ensure that your manuscript meets PLOS ONE's style requirements, including those for file naming. The PLOS ONE style templates can be found at | *PLOS ONE”s style guide was followed.* |
|  | Please ensure that you include a title page within your main document. | *Included* |
|  | If there are no restrictions, please upload the minimal anonymized data set necessary to replicate your study findings as either Supporting Information files or to a stable, public repository and provide us with the relevant URLs, DOIs, or accession numbers. | *Data link to this article is uploaded.* |
|  | To address above suggestion, I suggest to adjustments in title, results interpretation, and implications in conclusions section. Also, clearly mention in the methodology about the limitations of the study, i.e. non-representative, and have weak external validity (cannot be generalized for rural Bangladesh, even for rural borrowing women of Khulna Distt.). | *A section named ‘limitations of the study’ has been added bottom (page 10) in the methodology part. This addresses the limitations on non-representativeness of the location and microfinance recipients over the country. This should resolve the suggestions that has been made by reviewers and the editor.* |
|  | | |
| Reviewer #1 | 1. The citations format allows to mention the author name and respective governments are considered the author of all the official reports; rather mentioning the name of organization. For example, “Microfinance Regulatory Authority, 2018 at Pg-8”. | *We used the citation format suggested for Plos One articles. I think this resolve this issue. Nevertheless, the example given by #Rev 1 is taken from website of microfinance regulatory authority of Bangladesh thus it appeared without any specific author’s name.* |
|  | 2. The specified characteristics defined for the baseline to identify the informants of this study may lead to create biases as well as rigid sample and in the presence of such sample it becomes difficult to generalize the results for the  entire women borrowing community. | *The term ‘baseline’ has been changed to ‘criteria’ at page 9* |
|  | 3. Because of the fact that the study based upon the primary information and thus it is imperative to employ suitable statistical techniques i.e. Cronbach's Alpha, to examine the reliability of data. | *The quantitative data was included in this paper only to provide background context to the findings of the qualitative data presented from which findings were derived. Therefore, we used only simple statistics to describe the cohort of women interviewed rather than requiring any Higher statistical methods. We believe that the rich qualitative data presented which is the focus of the article and which the discussion and findings is based on means that higher-level statistics are not necessary for the quantitative analysis.* |
|  | 4. In the presence of such a rich information, the use of regression analysis may be a good addition and also more  interesting; rather using the simple percentages. Hence, it is suggested to use an appropriate econometric model i.e. Logit or Probit, to empirically determine the facts & figure for the policy derivation. |  |
|  | 5. No doubt the study is very interesting, but it lacks the suggested remedial measures to overcome this issue. | *The suggestions are very important, and we took the suggestions made by the reviewer #1 seriously. We tried to improve the paper according to the suggestions made and provided explanation in few areas where we have different opinion.* |
|  | | |
| Reviewer #2 | 1. Page 2 Microfinance has been widely recognized as an ideal type of aid and development intervention for enhancing women’s financial empowerment by increasing women’s income, financial contribution to household and financial decision-making power in the family (Ackerly, 1995; R. Amin & Becker, 1998; Assassi, 2009; Duflo, 2012; Grameen Bank,  2011; Khandker, 1998).  - Have any relevant publications appeared more recently? It would be worth a quick look and give latest references as  well. | *Latest references (Kaur, 2017; Sultana et al., 2017) has been added in Page 3* |
|  | - Recommended proofing to help to correct minor errors.  In this paper, we define patriarchy as “a system of power in which male privilege and superiority over women are manifest, institutionalised, and self-reproducing across a society as a whole” (Shepherd, 2019, p. 116). In this paper, we define patriarchy as “a system of power in which male privilege and superiority over women are manifested, institutionalised, and self-reproduced across a society as a whole” | *Some further review has occurred, and This correction has been made in page 4* |
|  | - The introduction part is nicely woven. It mentions “This mixed-method, multidisciplinary study examines the impact of gender norms on the financial empowerment of women recipients of microfinance in rural Bangladesh.” However, it doesn’t explicitly explain what is the objective of the study. In the same paragraph authors better add what is expected from this study, expected study implications. | *The objective ‘Relevant literature and our theoretical proposition add objective of this study to see how the microfinance borrowing women adjust financial dealings with the entranced patriarchy and its driven gender norms and practices’ has been added as suggested by reviewer#2 on page 4* |
|  | - This methodology and result sections are not necessary in Intro section.  To explore the issues above, we conducted 331 surveys and 33 in-depth interviews with women receiving microfinance and their husbands in the Dumuria sub-district in the southern region of Bangladesh. Bangladesh is globally recognised as the home of microfinance since the mid- 1970s (Yunus & Jolis, 1998). We found that women’s financial empowerment  is significantly undermined by entrenched gender norms and customary gender practices, regulated by an ontology of patriarchy. We argue that it is important to consider how women receiving microfinance and men internalise gender norms about finance and financial participation and that this is important to understand their financial empowerment  outcomes. | *This paragraph has been deleted as the reviewer #2 suggested. See page 5* |
|  | Gender socialisation and performance in families  - What is Purdah? It should be explained when used for the first time (first use) in the manuscript. In the current  sentence “Social institutions such as marriage (Delphy, 1984), purdah (White, 1992),…..” it is unclear. Please rearrange or rewrite the sentence. | *This comment of reviewer #2 has been addressed and the paragraph is re-organized with explanation of purdah is given. See at page 7* |
|  | This may present a gap in current knowledge, since the existing evidence presented in this section of the paper indicates that how women and men learn, perform and (re)produce gender norms in Bangladesh is influenced by financial factors; a woman receiving microfinance assistance and her spouse might be performing crucial roles about gender they have learned through the gender socialisation process, which may both influence and be strengthened by microfinance loan management and decision-making.  - Present or May present? If authors are presenting the research/knowledge gap, they should be sure about that, not  “may present a gap”. Long sentences like this one should be avoided. It is hard for readers to follow. Such long sentences  need rechecking/revision throughout the manuscript. | *The correction has been made, as ‘may’ is deleted and the sentence has been split (see at page 8)*  *“The evidence presented in this section shows how women and men learn, perform and (re)produce gender norms in Bangladesh and if this is affected by financial factors and constitutes a new approach generating new knowledge in the field of microfinance. The gender socialisation process can either be further entrenched by microfinance loan management and decision-making processes. The processes of change advocated for cannot be assumed with changes to gender roles and socialization practices.”* |
|  | Methodology  It is about how you collected data. So, it should be “this study collected primary data in southern Khulna,……” It’s not “this study presents….” in the methodology. It needs some language twists. | *Correction has been made in methodology section. See page 9.* |
|  | This study focuses mainly on women’s perceptions and experiences of microfinance in Bangladesh.  - As I understood, the study is performed in Dumuria sub-district of southern Khulna city. Does the study in this particular area represent the whole Bangladesh? I think the situation between city and rural Bangladesh must be different too. Can the result be generalized for whole Bangladesh? If not, then it should be revised accordingly. | *Correction made at page 9-10*  *,,, In line with #Rev 1 comments a section on limitations of the study and its non-generalizability has been added* |
|  | - The baseline for identifying informants of this study specified some characteristics: i) they had to be current female microfinance borrowers; ii) they should have at least two living children (one male child and one female child) up to 15 or more years old, and; iii) the women have or had at least one surviving brother (at least 15 years of old) while growing up  in the family home. The baseline (better to say criteria)… This sentence needs rewriting to improve the flow. Thorough editing is essential  help to improve the quality, readability of the paper. | *Baseline has been changed to criteria and the sentence is rewritten to improve the flow. See at page 9*  *The criteria for identifying informants in this study included: i) they had to be current female microfinance borrowers; ii) they should have at least two living children (one male and one female) and; iii) the women have or had at least one surviving brother. The criteria…* |
|  | We assume that a mother who had grown up with a brother(s), and have at least a son (or more) and a daughter (or more), would provide more comparative, valid and reliable data on household gender norms and its practices than a mother who had no brother or having either son(s) or daughter(s).  - I think both gives valid and reliable data but it’s the matter of different perspective that you are looking for from the earlier one for comparative analysis. | *I think, yes, the perspective is important. However, we used these criteria to understand better in our study.* |
|  | - Is there any reason why only 10% of the survey sample was chosen for in-depth interviews? | *We have deleted the 10% of the survey sample from page 9. However, to explain reviewer’s query, during the long field work we assumed that gathering at least 10% of the 331 survey informant’s data for our qualitative data would help us to provide representativeness of the sample.* |
|  | Is the semi-structured interview guide/schedule included along with this paper? That will be helpful for the readers to understand what kind of questionnaire is used for particular data collection. How long did the survey and in-depth interview took in average for study participants? | *The survey has been included.*  *This sentence has been added (see page 10):* *The survey took around 30 to 40 minutes and the in-depth interviews both took 45 to 60 minutes on average.* |
|  | - Methodology lacks details on data analysis. How the collected data are analyzed? E.g. Simple statistics, and what else??  Is it possible to do any statistical analysis??? | *The data processing and analysis for both quantitative and qualitative data has been included in page 10.*  *In addition, as we explained before, the quantitative data was included in this paper only to provide background context to the findings of the qualitative data presented from which findings were derived. Therefore, we used only simple statistics to describe the cohort of women interviewed rather than requiring any Higher statistical methods. We believe that the rich qualitative data presented which is the focus of the article and which the discussion and findings is based on means that higher-level statistics are not necessary for the quantitative analysis.* |
|  | Findings/Results  Overview of loan patterns and gendered decision-making  It will be effective to present the quantitative data/results in bar diagrams than in words. | *Graphical chart /bar diagram has been added in page 12, 13 and in 15.* |
|  | Purdah  However, Hossain and Kabir (2001) Agarwal (1997) argued that one-way purdah norm has been challenged by rural women is by microfinance participation.  - One-way purdah norm has been challenged by rural women is by microfinance participation. I agree but in overall, I think educating women (their access and continuity to (school and higher) education) is essential to change the purdah norm and control of male-counterparts in microfinance participation/decision-making. How education may affect gendered decision making, inter-generational reproduction of the gendered division of labour in future needs to be discussed. | *We agree with your argument of the importance of education to challenge purdah restriction. Hossain and Kabir (2001), Agarwal (1997) argued that purdah restrictions are strong in rural Bangladesh and microfinance participation and IGAs would minimize these practices. However, we argued that purdah restrictions are dictated by culture practiced for generations. therefore, changing purdah norm requires structural changes of cultural practices. Education could be one of the significant ways to foster these changes. We also see that our recipient women were mostly less educated. Hence this study findings would be useful to add education in the scope for future researchers to examine purdah norm with education. We added the importance of studying education and purdah for future researchers in page 23.* |
|  | Discussion  The current study challenges some of the main findings of scholarly literature investigating microfinance in Bangladesh, such as Mahbub (2001), M. M. Pitt, Khandker, and Cartwright (2003), S. R. Schuler, Hashemi, & Riley (1997) and Sharma (2008), who state that women’s microfinance participation improves their mobility and their participation in the market.  - It may be- women’s microfinance participation has improved their mobility and their participation in the market in some  other areas but not in southern Khulna. How about this? | *This is a good suggestion that we included in our text in page 23.* |
|  | These scholars showed that in Bangladesh, gender norms governing women’s mobility is strictly observed. - Is it throughout the country or only in rural areas? | *Women’s gender restrictions are common in Bangladesh, but the situation is worst in rural areas compare to urban areas. However, as our research is conducted in Rural Bangladesh, so we focus mostly to refer rural Bangladesh. Correction has been made in page 23.* |
|  | Importantly it also shows the lack of impact of microfinance interventions on patriarchal beliefs and practices in Bangladesh.  - Is this just because of purdah. I think it is largely because of no interventions in the education sector for women. If education is improved then it will improve the purdah norms and others consequently. This should be discussed if it’s within the scope of this study. | *As we mentioned early that education is important for fighting purdah restriction, but it is not currently been covered by this article’s scope.* |
|  | Therefore, women’s financial empowerment will require a more expansive intervention than microfinance participation: providing access to money alone cannot financially empower women.  - Again, I think it is largely because of no interventions in the education sector for women. If women are educated then they will overcome the gender and purdah norms/practices. Education will automatically empower women and their decision-making power consequently. This should be discussed if it’s within the scope of the study. | *We included text on education for future research scope. Please see page 23-24.* |
|  | Conclusion  Although this study did not intend to provide a fully representative picture of the perceptions of different microfinance recipient groups of women in the nation of Bangladesh, the responses and experiences explained here are so consistent that they may be important for comparing results of how different microfinance recipient groups experience these important issues.  - Authors must revise the earlier section in the similar way to reflect that it is not a full representative picture of the  whole country. As of now, in earlier sections, authors talk about whole Bangladesh. So, it is confusing. See my comments above. | *A section on limitation of the study has been added bottom in the methodology part. This addresses the limitations on non-representativeness of the location and microfinance recipients over the country. I think this part solve the wise suggestion that has been made by reviewers and the editor.* |
